# Roles of Nursing in the Management of Geriatric Cardiovascular Diseases

**DOI:** 10.3389/fmed.2021.682218

**Published:** 2021-09-08

**Authors:** Chunzhi Zhang, Congling Xiang, Xin Tian, Jun Xue, Gengxu He, Xueliang Wu, Zubing Mei, Tian Li

**Affiliations:** ^1^Department of Thoracic and Cardiovascular Surgery, First Affiliated Hospital of Hebei North University, Zhangjiakou, China; ^2^Department of Cardiology, Xinchang Hospital Affiliated to Wenzhou Medical University, Xinchang, China; ^3^Department of Cardiology, Traditional Chinese Medicine Hospital of Shaanxi Province, Xi'an, China; ^4^Department of Vascular Surgery, First Affiliated Hospital of Hebei North University, Zhangjiakou, China; ^5^Department of Anorectal Surgery, Shuguang Hospital, Shanghai University of Traditional Chinese Medicine, Shanghai, China; ^6^School of Basic Medicine, The Fourth Military Medical University, Xi'an, China

**Keywords:** cardiovascular disease, geriatrics, nursing management, heart failure, cardiac rehabilitation

## Abstract

The nursing field occupies the largest secion of the cardiovascular healthcare services. Despite this, the roles of nursing within the cardiovascular healthcare system has not been well displayed. The authors searched PubMed and Embase (between January 1, 1950, and June 17, 2021) and created a narrative review of recent publications regarding the role of nursing in the management of geriatric cardiovascular disease (CVD). Patients with geriatric CVD, which includes mainly myocardial ischemia and heart failure, were enrolled. Nursing can improve the outcomes of myocardial ischemia and heart failure. It plays a pivotal role in the recovery, rehabilitation, and outcomes of geriatric CVD, especially for chronic heart diseases. Taken together, this paper compiled is focused on the current status of cardiovascular nursing and may facilitate future treatment and rehabilitation in geriatric CVD.

## Introduction

Cardiovascular disease (CVD) remains a primary cause of mortality for people all over the world (1). According to statistical data from the American Heart Association (AHA), the age-adjusted prevalence of all types of heart disease was 10.6%. In 2017, CVD-induced deaths are about 17.8 million globally. The CVD-induced mortality is 219.4 per 100,000 in 2016. CVD has become a primary public health problem all over the world—far beyond cancer and unintentional injuries. Notably, 840,678 deaths from CVD were registered in the United States in 2016, which may contribute to significant economic and health burdens worldwide (2). According to the World Health Organization (WHO)'s World's Nursing Report 2020, there are over 27.9 million nurses globally, accounting for more than half (59%) of the number of medical workers in the world. Among them, 19.3 million are professional nurses (69%), and 6 million (22%) are professional nurse assistants (3).

In the nursing work of geriatric CVD, effective prevention of risk factors in nursing work is of great clinical significance to improve the quality of nursing and the early rehabilitation of geriatric CVD patients. As nurses have the most contact time with patients, they should not only be familiar with the characteristics of elderly cardiovascular disease, but they also acquire knowledge of the mental aspect of nursing, considering the social roles of the elderly.

**Figure 1:**
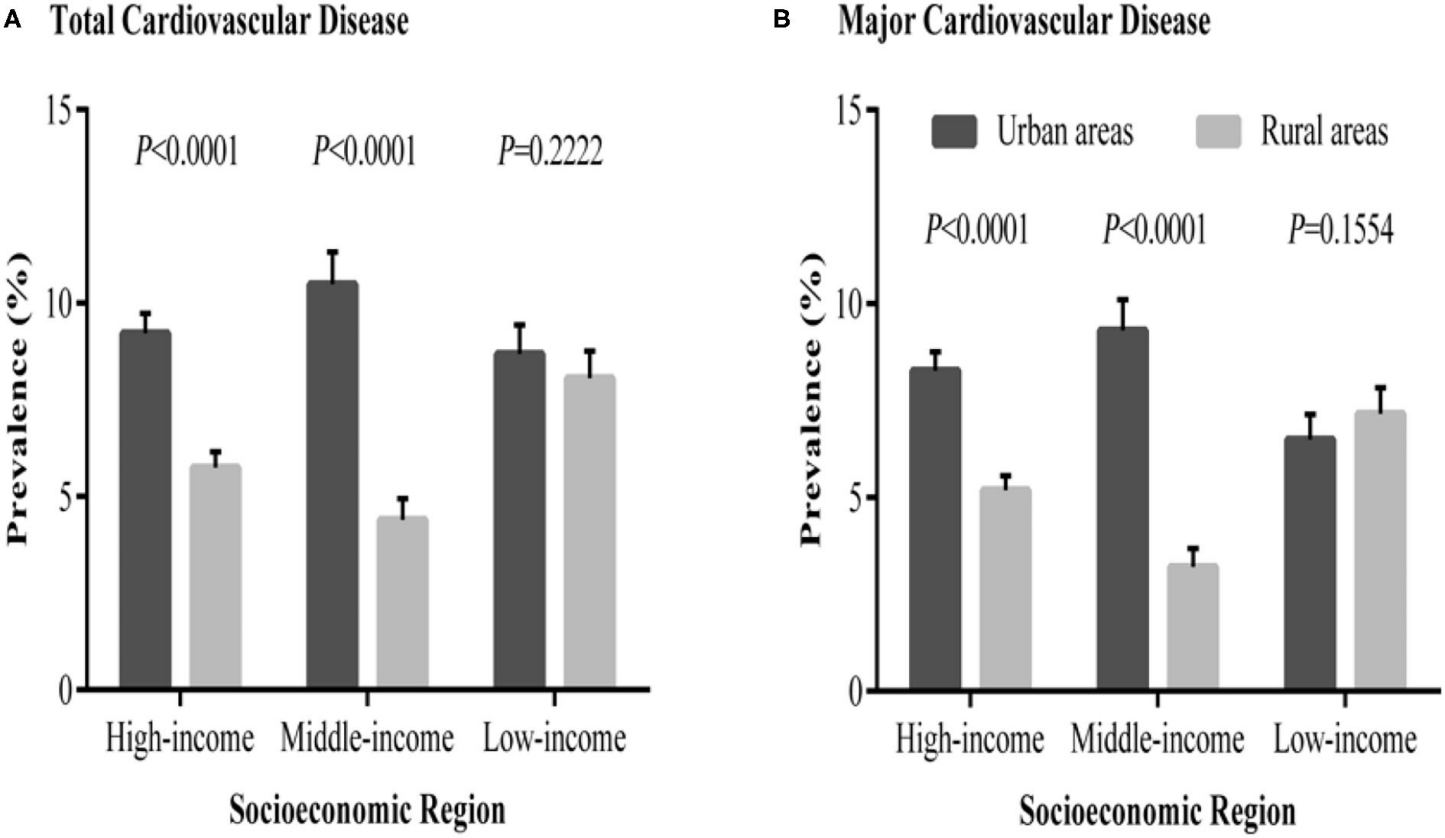
Prevalence of total and major cardiovascular diseases in urban and rural communities in different socioeconomic regions of China. **(A)** The distribution of total cardiovascular disease prevalence. **(B)** The distribution of major cardiovascular disease prevalence (8).

China is now undergoing unprecedented economic growth and globalization, and this has resulted in an increase in the prevalence of chronic conditions, particularly CVD (4). CVD makes up 40% of all deaths in China, and it is thus the highest single cause of mortality. It is estimated that there are 290 million CVD patients in the world, which has created serious challenges for the improvement of public health. However, the population tends to have a low rate of awareness of CVD as well low popularization rates of CVD prevention in both urban and rural districts in China (5–7) (Figure 1). The world has witnessed huge changes in health services in China since 1978. Peking Union hospital trained the first nurses with bachelor's degrees (4). Cardiovascular nursing is dedicated to the nursing service and self-management of patients with CVD. It plays a pivotal role in the recovery, rehabilitation, and outcomes of geriatric CVD, especially chronic heart diseases. Nurse occupies the largest parts of cardiovascular healthcare services, whereas the roles of nursing within the cardiovascular healthcare system have not been highlighted well. There is therefore an emerging call for enhancing the roles of nursing in the whole process of CVD, especially geriatric CVD.

This narrative review is aimed at discussing the roles of nursing in the management of geriatric CVD. Firstly, we introduce the background of CVD and cardiovascular nursing, including the epidemiology and therapeutics of CVD as well as the current status of nursing. Secondly, we review the roles of nursing in the management of CVD such as heart failure and myocardial ischemia. Thirdly, we talk about the concept of nursing education. Finally, we discuss home- and clinic-based nursing, cardiac rehabilitation, and individual care. This article highlights the recent advances and provides an elaborate picture of cardiovascular nursing, which might be helpful in nursing care and therapy for CVD.

## Roles of Nursing in the Management of Geriatric CVD

### Heart Failure

The symptoms of heart failure are characterized by shortness of breath, fluid retention, poor oxygen delivery, and impaired function of the heart itself (9). The prevalence of heart failure is increasing with age. There are 6.2 million adults ≥20 years of age who experienced heart failure (HF) between 2013 and 2016 in the United States of America (USA) (9).

In total, 127 patients with reduced ejection fractions (EFs) were prospectively allocated to standard care or intervention programs (1:2) at random from 2011 to 2013. Ortiz-Bautista et al. (10) found that readmissions for HF were reduced and life quality is significantly improved in the intervention group. A total of 59 patients were divided into three groups: collaborative management, self-management education, and usual care. The results show that the quality of life (QOL) score is improved in collaborative management compared to usual care at 18 and 24 months. Collaborative management may increase psychosocial status for HF patients and prevent HF-related rehospitalization (11).

HF may influence a patient's quality of life to a great extent. Rorth et al. (12) found that HF-led hospitalization is associated with increased nursing home admissions and domiciliary support. In total, 51 HF patients were enrolled and thereafter received adaptive servo-ventilation for trial evaluation. Results show that this treatment may improve the QOL of HF patients (13).

### Myocardial Ischemia

Myocardial ischemia is referred to the insufficient flow of the coronary artery and is an early pathological stage of CVD, such as myocardial infarction and acute coronary syndrome (14). After the 12-month intervention, health-related QOL, cardiac risk factors, and health behaviors of the participants undergoing percutaneous coronary intervention (PCI) greatly improved compared to controls (15).

Psychological nursing used computed tomography angiography (CTA) technology-based nursing methods for patients with CHD. Systolic blood pressure, heart rate, diastolic blood pressure, self-rating anxiety scale, and the self-rating depression scale scores of patients in the observation group were notably lower than controls (16). Seidl et al. found that the cost-effectiveness of nurse-based case management is cost-neutral and contributes to a great improvement in health status among survivors. It was associated with higher quality of adjusted life years and lower costs in elderly patients with myocardial infarction (17). Moreover, Kirchberger et al. (18) reported that nurse-based management among elderly patients with acute myocardial infarction (AMI) improves functional status and malnutrition.

## Nursing Education

### Education of Nurses

There is no standardized protocol for HF education programs. Researchers and nursing staff are still exploring the best form of education. Breathett et al. added a novel tablet application to nurse practitioner (NP) education. They found that the addition of novel tablet applications may contribute to better patient satisfaction and teaching effects for HF patients (19). Addiction of nursing knowledge of electrocardiograms of myocardial ischemia and myocardial infarction will increase the mean score on the pretest in an urban hospital of the Midwest and improve patient outcomes (20).

### Nurses' Education for Patients

Cardiosurgery nurses performed preoperative education for inpatients. They found that nurse-led preoperative education is helpful for reducing preoperative anxiety and postoperative cardiovascular/mortality complications, whereas it fails to reduce the possibility of second admissions and length of admission (21). Awoke and colleagues found that a nurse-led HF inpatient hospital education is suggested to improve reduce the 30-day possibility of second admission and self-care ability (22).

## Perspectives

### Home and Clinic-Based Nursing

Maru et al. enrolled 611 patients with subclinical cardiovascular diseases (without chronic heart failure) (23). In total, 13 patients recovering from acute coronary syndromes (ACS) were interviewed by telephone, and seven nurse mentors completed a survey after completing the program in Australia. There were positive signs that the program influenced patients' decisions to change unhealthy lifestyle behaviors. Outcomes highlighted both rewards and barriers associated with telephone-led patients follow-up visits (24). In Sweden, ~40% of identified HF cohort was enrolled in a nurse-led HF clinic (25). In the USA, nurse-led clinic-based nursing was helpful in reducing 30-day second admission by ~9% (26).

### Cardiac Rehabilitation

Cardiac rehabilitation improves prognosis after an acute AMI, however, the optimal method of implementation is unknown. In total, 217 patients after an AMI received tailored care through a nurse-led cardiac rehabilitation program, and this achieved better control of total cholesterol, low-density lipoprotein (LDL) cholesterol, and systolic blood pressure. This suggests that a tailored, nurse-led cardiac rehabilitation program improves risk factor management in post-AMI patients.

## Conclusions and Implications

The elderly are usually faced with several major comorbidities/risk factors of CVD (2). The current problems of nursing in the management of elderly cardiovascular diseases still remain and are obstacles in the prevention and post-onset management. CVD is the first cause of disability and death in the elderly over 60 years old. Firstly, due to the particularity of the elderly group, in the process of nursing for geriatric CVD patients, we must pay attention to their psychological changes, and actively take measures to deal with their psychological situation (27). Secondly, the elderly often have a decreased understanding and learning ability, which causes difficulties for health education. The penetration degree of nursing health education is lacking in elderly CVD patients (28). There is a gap in nursing care for geriatric CVD patients. Thirdly, the importance of nursing has not been well-displayed for geriatric CVD compared with medical activity (consulting a cardiologist). Patients may seek help from doctors more than nurses.

In the nursing work of geriatric CVD, effective prevention of risk factors in nursing work is of great clinical significance to improve the quality of nursing and the early rehabilitation of geriatric CVD patients. As nurses who have the most contact time with patients, they should not only be familiar with the characteristics of elderly cardiovascular disease, but they should also have knowledge of mental nursing, considering the social roles of the elderly. Nurses should strengthen the study of etiology, pathogenesis, drug indications, and adverse reactions of CVD to improve the levels of professional technology. They should also actively communicate with patients, establish a relationship of mutual trust and understanding, and carry out targeted psychological nursing, illness nursing, diet nursing, and discharge guidance according to elders' different conditions and personality characteristics so as to improve the quality of nursing and the recovery rate of geriatric CVD patients.

Though this narrative illustrates the most recent advances of nursing in the management of geriatric cardiovascular disease, there remain limitations and hope that further research/works can improve. Firstly, the authors failed to make a systematic review (evidence-based search) and only make a narrative review summary. Secondly, the authors failed to provide a comprehensive review: parts of the content are not analyzed in great detail, and this is possibly due to the length limitation of this narrative article and journal requirements.

CVD remains the main killer for people throughout the world (14, 29, 30). At the beginning of this manuscript, we introduce the background of CVD and nursing, followed by roles of nursing in the management of CVD such as HF and myocardial ischemia. Secondly, we discuss how nursing aims to provide a reference for cardiovascular nursing. Eventually, we analyze home- and clinic-based nursing, cardiac rehabilitation, and self-care. Taken together, this paper compiled here is devoted to the current status of cardiovascular nursing and may facilitate future research and progress in CVD.

## Author Contributions

TL, XW, and ZM: conceptualization. CZ and CX: writing – original draft. XT, JX, GH, and TL: writing – review and editing. All authors contributed to the article and approved the submitted version.

## Funding

This study was supported by Key Research Project of Medical Science of Health and Family Planning Commission of Hebei Province (ZD20140476).

## Conflict of Interest

The authors declare that the research was conducted in the absence of any commercial or financial relationships that could be construed as a potential conflict of interest.

## Publisher's Note

All claims expressed in this article are solely those of the authors and do not necessarily represent those of their affiliated organizations, or those of the publisher, the editors and the reviewers. Any product that may be evaluated in this article, or claim that may be made by its manufacturer, is not guaranteed or endorsed by the publisher.
