# Erratum: Roles of Nursing in the Management of Geriatric Cardiovascular Diseases

**DOI:** 10.3389/fmed.2021.820659

**Published:** 2021-12-10

**Authors:** Frontiers Production Office

**Affiliations:** Frontiers Media SA, Lausanne, Switzerland

**Keywords:** cardiovascular disease, geriatrics, nursing management, heart failure, cardiac rehabilitation

Due to a production error, the corresponding authors were listed in the incorrect order. They should be listed as follows:

Tian Li

fmmult@foxmail.com

Zubing Mei

herrmayor@126.com

Xueliang Wu

wuxl@hebeinu.edu.cn

The publisher apologizes for this mistake. The original version of this article has been updated.

